# Diphtheria Toxin A-Resistant Cell Lines Enable Robust Production and Evaluation of DTA-Encoding Lentiviruses

**DOI:** 10.1038/s41598-019-45481-9

**Published:** 2019-06-20

**Authors:** Margaret J. Lange, Terri D. Lyddon, Marc C. Johnson

**Affiliations:** 1 Department of Molecular Microbiology and Immunology, University of Missouri, Columbia, Missouri USA; 2 Bond Life Sciences Center, University of Missouri, Columbia, Missouri USA; 3 Present Address: Department of Molecular Microbiology & Immunology, Bond Life Sciences Center, University of Missouri, Columbia, MO 65211 United States

**Keywords:** Genetic vectors, Genetic engineering

## Abstract

Suicide genes have been widely investigated for their utility as therapeutic agents and as tools for *in vitro* negative selection strategies. Several methods for delivery of suicide genes have been explored. Two important considerations for delivery are the quantity of delivered cargo and the ability to target the cargo to specific cells. Delivery using a lentiviral vector is particularly attractive due to the ability to encode the gene within the viral genome, as well as the ability to limit off-target effects by using cell type-specific glycoproteins. Here, we present the design and validation of a diphtheria toxin A (DTA)-encoding lentiviral vector expressing DTA under the control of a constituitive promoter to allow for expression of DTA in a variety of cell types, with specificity provided via selection of glycoproteins for pseudotyping of the lentiviral particles. DTA exerts its toxic activity through inhibition of eukaryotic translation elongation factor 2 (eEF2) via adenosine diphosphate (ADP)-ribosylation of a modified histidine residue, diphthamide, at His715, which blocks protein translation and leads to cell death. Thus, we also detail development of DTA-resistant cell lines, engineered through CRISPR/Cas9-mediated knockout of the diphthamide 1 (DPH1) gene, which enable both robust virus production by transfection and evaluation of DTA-expressing virus infectivity.

## Introduction

Suicide genes encode proteins that are toxic to host cells at very low levels of expression, and have been widely investigated for their utility as therapeutic agents and as tools for *in vitro* negative selection strategies^1–3^. One commonly used suicide gene is the catalytic diphtheria toxin fragment A gene (DTA). Diphtheria toxin (DT) is a 62 kDa protein secreted by the gram positive bacillus, *Corynebacterium diphtheria*^4,5^. The A fragment inhibits eukaryotic translation elongation factor 2 (eEF2) through adenosine diphosphate (ADP)-ribosylation of a modified histidine residue, diphthamide, at His715, which blocks protein translation and leads to cell death^4–7^. Biosynthesis of diphthamide consists of stepwise modifications to His715 by a series of proteins, Dph1-7^7^. Interestingly, disruption of diphthamide biosynthesis does not affect protein translation or cell viability^7–9^.

Despite the obvious experimental and therapeutic potential of suicide genes, their inability to cross biological membranes and enter cells remains a significant barrier to their use. Several methods for delivery of suicide genes have been tested, including conjugation to cell penetrating peptides (CPPs)^10^, encapsidation of the protein into viral like particles (VLPs)^11,12^, delivery via liposomes and nanoparticles^13–15^, and use of viral vectors^16–21^. Two important considerations for each method of delivery are the quantity of delivered cargo and the ability to target the cargo to specific cells to avoid nonspecific cell death. Delivery using a lentiviral vector is particularly attractive due to the ability to encode the gene within the viral genome, which is then integrated into the host genome. To achieve targeted expression, many laboratories have used tissue-specific or inducible promoters^18,21–24^. These strategies allow for expression of the transgene only in cells where the tissue-specific promoter is functional or that are treated with a drug for induction of the inducible promoter. However, for use in a variety of different cell types or experimental systems, the vectors must be re-engineered and the conditions for induction optimized. Here, we set out to design and validate a DTA-encoding lentiviral vector that expresses DTA under the control of a constituitive promoter to allow for expression of DTA in a variety of cell types. Utility of this DTA-expressing vector could apply to a variety of experimental strategies, such as those employing genome-wide CRISPR/Cas9 screening to identify cells resistant to infection by the lentiviral vector, those examining mutagenized envelope glycoproteins to ascertain compatibility with a variety of cell types, or those to identify yet unknown envelope glycoprotein receptors and co-receptors. To produce and validate our vector, we also engineered two DTA-resistant cell lines using CRISPR/Cas9-mediated knockout of the DPH1 gene to allow both robust production of DTA-encoding viruses, as well as validation of DTA-specific effects upon virus transduction of target cells. Using this system, we demonstrate that DTA-encoding lentiviruses are capable of inducing cell death in DTA-susceptible cells, as well as targeting specific cells in a mixed population via use of specific viral glycoproteins for pseudotyping of the lentiviral particles by transfection of producer cells.

## Results

### Design of lentivirus encoding the diphtheria toxin a gene

**Figure 1:**
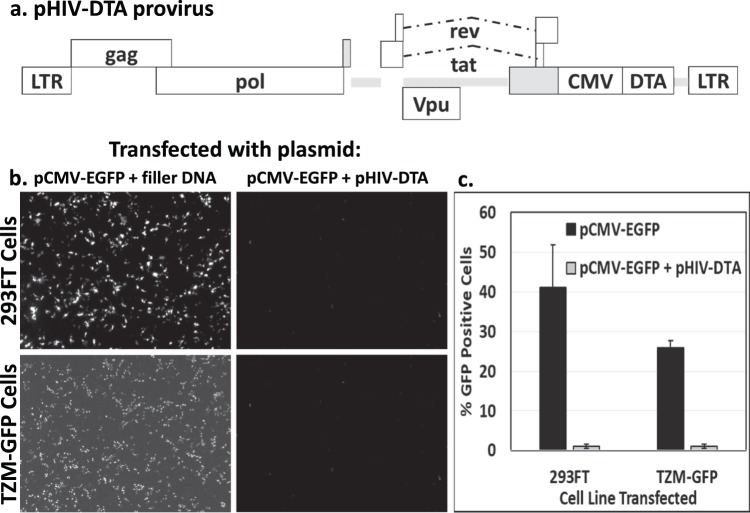
A proviral plasmid encoding DTA blocks reporter EGFP translation in 293FT and TZM-GFP cells. (**a**) Schematic representation of the DTA-encoding lentiviral vector, pHIV-CMV-DTA. (**b**) Fluorescent images at 40x magnification of 293FT (top row) or TZM-GFP (bottom row) cells transfected with a combination of pCMV-EGFP and filler DNA (left column) or pCMV-EGFP and pHIV-CMV-DTA (right column). (**c**) Quantification of EGFP fluorescence in 293FT cells transfected as in (**b**) by flow cytometry. Experiments were performed at least three times in triplicate. A representative experiment is shown. Error bars represent standard deviation of triplicate transfections.

To effectively deliver DTA to target cells, we first engineered a lentiviral vector encoding DTA under control of the cytomegalovirus (CMV) promoter (Fig. 1a). The DTA-encoding lentiviral vector was derived from the HIV-1_NL4-3_-based plasmid, pHIV-CMV-EGFP. This proviral vector lacks the genes encoding *vif*, *vpr*, *vpu*, *nef*, and *env*, and has CMV immediate early promoter-driven EGFP in place of *nef*. For engineering of the DTA-encoding lentiviral vector, we replaced the EGFP gene with the gene for DTA. Because the proviral plasmid lacks the gene for env, a secondary plasmid encoding a viral glycoprotein must be used to produce infectious particles (pseudotyping).

### DTA inhibits protein translation in transfected and transduced cells

DTA inhibits eEF2 through ADP-ribosylation of a modified histidine residue, diphthamide, at His715, which blocks protein translation and leads to cell death^7^. To examine whether the DTA encoded by our proviral plasmid was functional, we assessed the ability of our constructs to block EGFP protein translation in 293FT (producer cells for production of lentiviral particles) and TZM-GFP (target HIV reporter cell line for lentiviral transduction) cells. The 293FT cells were chosen due to their high transfection efficiency and ability to produce high levels of lentiviral particles via transfection. The TZM-GFP cells were chosen as target cells due to the presence of a Tat-driven, HIV-specific GFP reporter that allows quantification of HIV-DTA transduction^25^. We co-transfected either 293FT (Fig. 1b, top row) or TZM-GFP (Fig. 1b, bottom row) cells with a combination of pCMV-EGFP with filler DNA (Fig. 1b, left column) or pCMV-EGFP with pHIV-CMV-DTA (Fig. 1b, right column). As shown in Fig. 1b, co-transfection of pCMV-EGFP with pHIV-CMV-DTA completely blocks EGFP protein translation (right column) as compared to co-transfection with pCMV-EGFP and filler DNA (left column). Thus, the DTA produced by transfection of our proviral plasmid is functional. Quantification of the transfection results is shown in Fig. 1c.

### Engineering DTA-resistant cell lines

**Figure 2:**
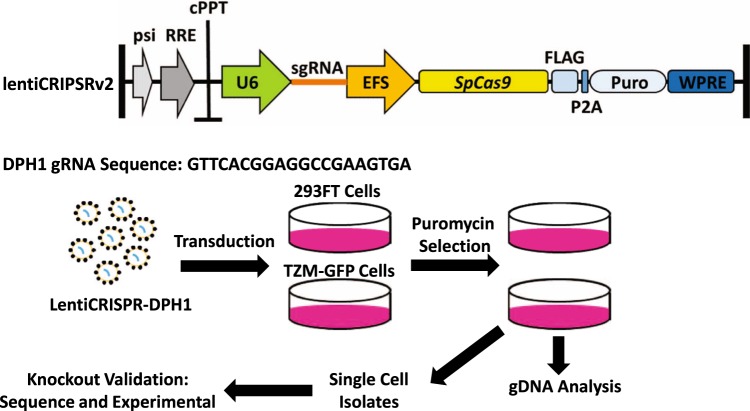
Engineering DTA-resistant cell lines using CRISPR/Cas9. Schematic representation of the lentiCRISPRv2 vector, guide RNA (gRNA) sequence specific to the DPH1 gene, and workflow for DPH1 knockout cell line generation. Black bars represent  long terminal repeats (LTRs). Psi = packaging signal, RRE = Rev response element, cPPT = central polypyrimidine tract, EFS = elongation factor short promoter, P2A = cleavage peptide, Puro = puromycin resistance cassette, WPRE = Woodchuck Hepatitis Virus posttranscriptional regulatory element.

As the DTA produced upon transfection of pHIV-CMV-DTA was functional and capable of inhibiting protein translation, producing infectious particles in cells susceptible to the effects of DTA was not possible. To overcome this problem, we set out to engineer a DTA-resistant producer cell line to enable production of lentiviral particles for efficient gene transfer to target cells. We also needed a method to titer DTA-encoding virus following virus production. Therefore, in addition to the DTA-resistant producer cell line, we also wanted to engineer a DTA-resistant target cell line to allow determination of viral infectivity and to ensure that any observed inhibition of protein translation or cell death was due to the expression of DTA rather than to an effect of the lentiviral vector. Several laboratories have demonstrated that DTA resistance can be achieved through blockade of various diphthamide biosynthesis proteins, either by expression of a dominant negative protein or by mutagenesis, without altering cellular viability^2,8,26^. Thus, to engineer our DTA-resistant cell lines, we chose to use the CRISPR/Cas9 system^27^ to knockout a key gene in the diphthamide biosynthesis pathway, DPH1^7,8,28^, in 293FT (producer cell line) and TZM-GFP (target cell line encoding an HIV reporter) cells. As illustrated in Fig. 2, we cloned a guide sequence targeting exon 1 of the DPH1 gene into the lentiCRISPRv2 vector, which encodes a puromycin resistance gene for selection. We used this vector to produce lentiviral particles, which were then used to transduce 293FT and TZM-GFP cells. Following transduction, modified cells were selected using puromycin, and validated for disruption of the DPH1 target by sequencing the gRNA target site.

### Knockout of DPH1 in 293FT and TZM-GFP cells prevents the DTA-induced block to protein translation

Following verification of disruption of the DPH1 target site, polyclonal DPH1 knockout populations were preliminarily tested for resistance to the DTA-induced block to protein translation by co-transfection of each polyclonal cell line with either pCMV-EGFP and filler DNA, or pCMV-EGFP and pHIV-CMV-DTA (data not shown). Once DTA resistance in the polyclonal cell populations was confirmed, we generated single cell isolates by limiting dilution. Twelve single cell isolates were screened by co-transfection of pCMV-EGFP and pHIV-CMV-DTA and scoring for EGFP expression (data not shown). A subset of clones with restored EGFP expression were subjected to analysis of DPH1 target site modification. The DPH1 target sequence, the relevant portion of the DTA gene sequence, the DTA validation primers, and the presence of the gRNA target sequence in the 293FT cells are shown in Fig. S1a–d, respectively. Insertions or deletions were identified using the CRISPR TIDE web tool^29^ and by alignment analysis of the sequence data. Most of the analyzed clones demonstrated heterozygous modification, with wildtype sequence remaining at one locus (data not shown). One monoclonal knockout line from each parental cell line demonstrated to exhibit complete modification (no wildtype sequence) was chosen for further experiments. For the 293FT-DPH1KO cells, the selected monoclonal cell line exhibited deletions at positions −8, −9, and −16, indicating a trimeric locus (Fig. S1e). The TZM-GFP-DPH1KO cells exhibited deletions at positions −28 and −34 (Fig. S1f).

**Figure 3:**
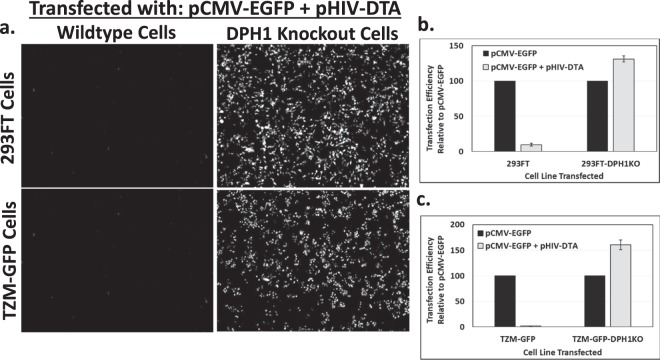
Knockout of DPH1 prevents the DTA-mediated block to EGFP protein translation in 293FT and TZM-GFP cells. (**a**) Fluorescent images at 40x magnification of 293FT (top left), 293FT-DPH1KO (top right), TZM-GFP (bottom left), and TZM-GFP-DPH1KO (bottom right) cells transfected with a combination of pCMV-EGFP and pHIV-CMV-EGFP. (**b**,**c**) Quantification of EGFP fluorescence in 293FT (**b**), 293FT-DPH1KO (**b**), TZM-GFP (**c**), and TZM-GFP-DPH1KO (**d**) transfected with either pCMV-EGFP and filler DNA (black bars) or pCMV-EGFP and pHIV-CMV-DTA (gray bars) by flow cytometry. Raw EGFP fluorescence values were normalized to the pCMV-EGFP + filler DNA transfection controls, and data is shown as transfection efficiency relative to the controls. Experiments were performed at least three times in triplicate. A representative experiment is shown. Error bars represent standard deviation of triplicate transfections.

To further validate the DTA resistance profiles of the selected monoclonal cell lines, we performed experiments to determine the level of DTA resistance. Parental and DPH1 knockout cell lines were co-transfected with either a combination of pCMV-EGFP with filler DNA or pCMV-EGFP with pHIV-CMV-DTA. As shown in Fig. 3, knockout of DPH1 in both the 293FT (Fig. 3a, top row) and TZM-GFP (Fig. 3a, bottom row) cells resulted in a complete restoration of EGFP protein expression in the presence of DTA. Interestingly, a slight enhancement of EGFP expression was observed after quantification of transfection efficiency by flow cytometry for both knockout cell lines (Fig. 3b,c). We have observed a similar enhancement of proviral plasmid transfection efficiency when co-transfected with other plasmids in other experimental systems, although we have not investigated the specific reason for this enhancement. The enhancement occurs regardless of the specific filler plasmid used (data not shown).

### Knockout of DPH1 in 293FT cells allows for robust production of DTA-encoding lentiviral particles

**Figure 4:**
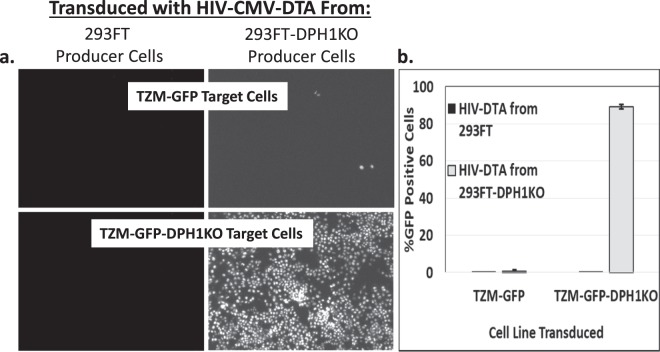
Knockout of DPH1 allows for robust production of DTA-encoding lentiviral particles. (**a**) Fluorescent images at 100x magnification of TZM-GFP (top row) and TZM-GFP-DPH1KO (bottom row) cells 48 hours post-transduction with VSV-G-pseudotyped HIV-CMV-DTA produced in 293FT cells (left column) or 293FT-DPH1KO cells (right column) transfected with a combination of pHIV-CMV-DTA (1000 ng) and pVSV-G (100 ng). (**b**) Quantification of EGFP fluorescence 48 hours post-transduction in TZM-GFP and TZM-GFP-DPH1KO cells. Virus was produced in either 293FT cells (black bars) or 293FT-DPH1KO cells (gray bars). Experiments were performed at least three times in triplicate. One representative experiment is shown. Error bars represent standard deviation of transductions from triplicate transfections.

Since DPH1 knockout cells demonstrated full restoration of EGFP protein translation in the presence of DTA, we next determined whether the 293FT-DPH1KO cells were capable of production of DTA-encoding lentiviral particles. As shown in Fig. 4, we observed robust production of lentiviral particles in the 293FT-DPH1KO cell line. The 293FT cell line produced little to no infectious particles (Fig. 4a, left column, and 4b), as expected, due to the ability of DTA to inhibit protein translation. Notably, lentiviral particles produced in the 293FT-DPH1KO cell line demonstrated high levels of EGFP expression in the TZM-GFP-DPH1KO cell line (Fig. 4a, bottom right, and 4b), but not the parental TZM-GFP cell line (Fig. 4a, top right, and 4b), further demonstrating the ability of DTA to block EGFP protein translation in non-DTA-resistant cells. Titers for HIV-DTA obtained from the 293FT-DPH1KO cell line were obtained by performing serial dilutions of virus-containing medium on TZM-GFP-DPH1KO cells and counting infectious foci, and consistently yielded 4–5 × 10^5^ infectious particles per mL.

### Enhanced DTA resistance and virus production in DPH1 knockout cells as compared to cells with an EF2 mutation

**Figure 5:**
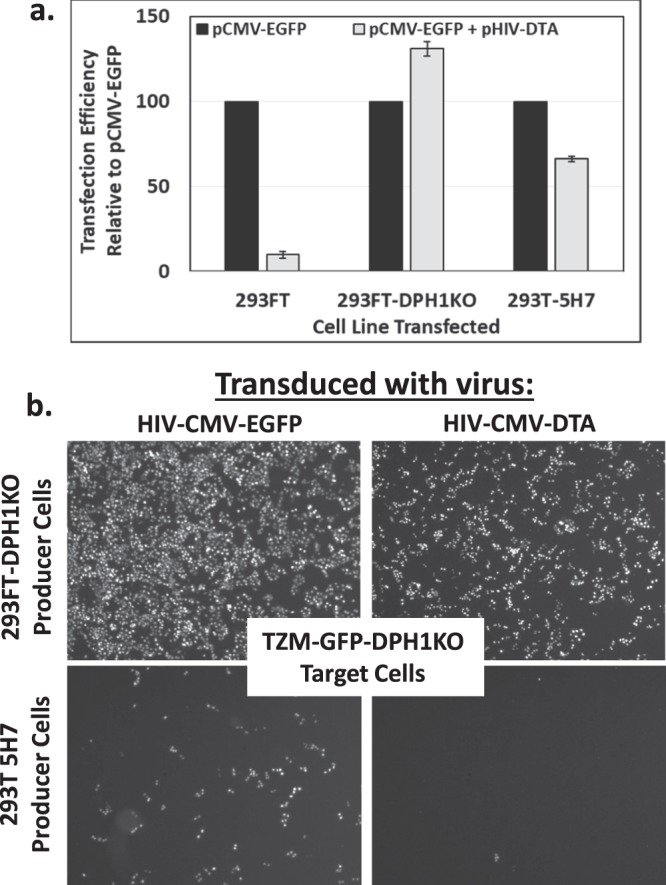
Knockout of DPH1 yields enhanced DTA resistance (**a**) and infectious virus production (**b**) as compared to mutation of EF2. (**a**) Quantification of EGFP fluorescence in 293FT, 293FT-DPH1KO, and 293T-5H7 cells 48 hours post-transfection with either pCMV-EGFP and filler DNA or pCMV-EGFP and pHIV-CMV-DTA by flow cytometry. Raw EGFP fluorescence values were normalized to the pCMV-EGFP + filler DNA transfection controls, and data is shown as transfection efficiency relative to the controls. Experiments were performed twice in triplicate. A representative experiment is shown. Error bars represent standard deviation of triplicate transfections. (**b**) Fluorescent images comparing transduction efficiency of TZM-GFP-DPH1KO cells transduced with parental virus, HIV-CMV-EGFP, produced in 293FT-DPH1KO or 293T-5H7 cells (left column) and HIV-CMV-DTA virus produced in 293FT-DPH1KO or 293T-5H7 cells (right column).

Previously, laboratories have addressed the problem of producing viral vectors encoding DTA by engineering DTA-resistant cell lines encoding a mutated eEF2 gene^30,31^. Thus, we wanted to compare the resistance levels of our 293FT-DPH1KO cell line with a cell line encoding a mutated EF2 gene. We obtained a commercially available cell line encoding a mutated eEF2 gene, 293T 5H7^18^. We found that while the 293T 5H7 cells demonstrated resistance to DTA upon co-transfection of pCMV-EGFP and pHIV-CMV-DTA, EGFP protein translation was not completely restored to levels observed by co-transfection of pCMV-EGFP and filler DNA (Fig. 5a).

We next compared the infectivity of viruses produced in 293FT-DPH1KO and 293T 5H7 cells. Both cell lines were co-transfected with a combination of either the parental proviral vector, pHIV-CMV-EGFP, or pHIV-CMV-DTA with VSV-G, and the resulting viruses were used to transduce TZM-GFP-DPH1KO cells (Fig. 5b). Robust particle production was observed for viruses produced in 293FT-DPH1KO cell lines (Fig. 5b, top row), while very low levels of viral production were observed for viruses produced in 293T 5H7 cells (Fig. 5b, bottom row). Since we observed complete restoration of EGFP expression and robust virus production in our 293FT-DPH1KO cell line, we performed future experiments with this cell line.

### Lentiviruses encoding DTA induce cell death in wildtype cells, but not DPH1 knockout cells

**Figure 6:**
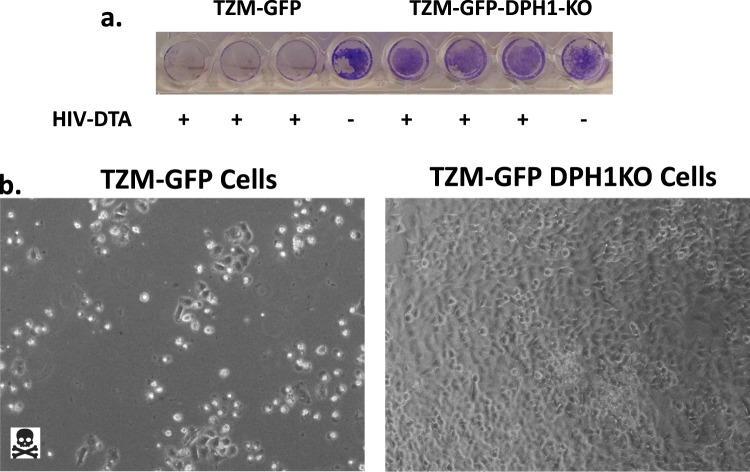
Lentiviruses encoding DTA induce cell death in wildtype cells, but not in DPH1 knockout cells. (**a**) Crystal violet staining of TZM-GFP and TZM-GFP-DPH1KO cells transduced in triplicate with HIV-CMV-DTA at an MOI > 1. Non-transduced controls are also shown. Crystal violet staining was performed 96 hours post-transduction. (**b**) Bright field images at 100x magnification of TZM-GFP (left) and TZM-GFP-DPH1KO (right) cells 96 hours post-transduction with HIV-CMV-DTA at an MOI > 1.

One important consideration for lentiviral-mediated delivery of DTA is expression level of the delivered cargo, as the DTA must be delivered in high enough quantity to induce cell death. To assess the ability of DTA delivered by our lentiviral vectors to induce cell death, we transduced both parental TZM-GFP and TZM-GFP-DPH1KO cells with a high MOI (>1) of HIV-CMV-DTA produced by transfection of 293FT-DPH1KO cells. As shown in Fig. 6, our lentiviral particles are able to induce significant cell death in the TZM-GFP cells, but not in the DTA-resistant TZM-GFP-DPH1KO cells.

### Lentiviruses encoding DTA can direct targeted cell killing

**Figure 7:**
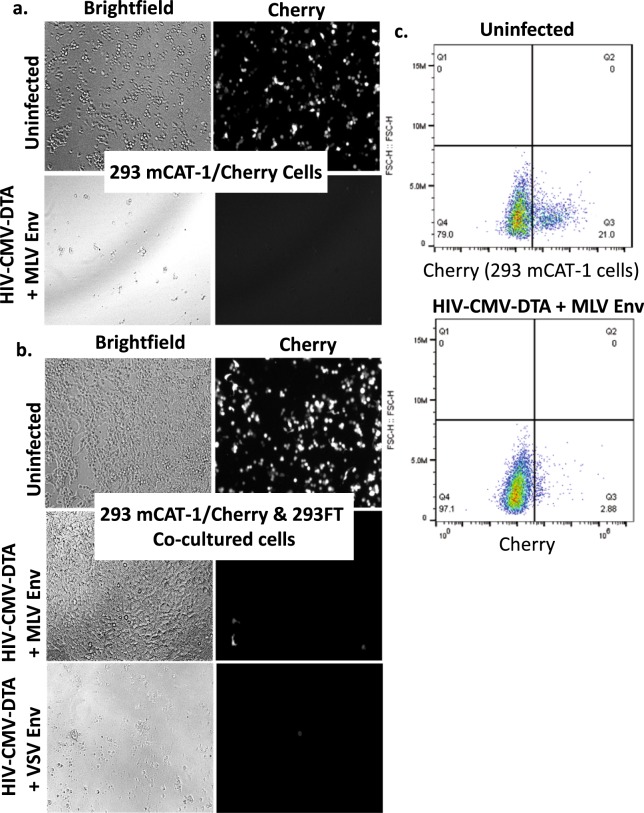
HIV-CMV-DTA-mediated, glycoprotein-targeted killing of 293 mCAT-1/Cherry cells in a mixed culture with 293FT cells. 293 mCAT-1 cells were transfected with a plasmid for expression of mCherry under the CMV promoter. 48 hours post-transfection, 293FT and 293 mCAT-1/Cherry cells were plated in a mixed culture at a 1:1 ratio by seeding 10,000 cells per well in 96-well plates. 293 mCAT-1/Cherry cells were also plated in a single culture at 10,000 cells per well in 96-well plates. Cells were infected or not infected with HIV-CMV-DTA pseudotyped with MLV Env (**a**,**b**) or VSV-G (b, bottom panel) at an MOI > 1. After 72 hours, cells were re-plated to allow detachment of dead cells and re-adherence of live cells. Bright field and fluorescent images at 100x magnification were taken 96 hours post-infection using an Olympus XI81 microscope. Remaining mCAT-1 cells expressing mCherry were also quantified by Flow Cytometry (**c**).

One additional benefit to the use of lentiviral vectors is the ability to limit off-target effects by using cell type-specific glycoproteins^32^. As proof of principle and to confirm the utility of our system for negative selection applications, we performed mixed culture experiments to determine whether HIV-CMV-DTA pseudotyped with the Ecotropic MLV envelope glycoprotein (MLV Env) could specifically target cells expressing the MLV Env receptor, mCAT-1. To allow for visualization of the two different cell populations, the 293 mCAT-1 cells were transfected with a plasmid for expression of mCherry prior to co-culture. As shown in Fig. 7, MLV Env-pseudotyped HIV-CMV-DTA transduction results in cell death for both single culture 293 mCAT-1 cells (Fig. 7a), and 293 mCAT-1 cells in co-culture with 293FT cells (Fig. 7b), as evidenced by a reduction in the Cherry-positive cell population. Transduction of the mixed culture with VSV-G-pseudotyped HIV-CMV-DTA, however, resulted in death of both cell populations (Fig. 7b, bottom panel). Notably, the 293FT cells in the mixed culture continue to proliferate (Fig. 7b). Quantification of the reduction in Cherry-expressing 293 mCAT-1 cells by flow cytometry is shown in Fig. 7c. Cells infected with VSV-G-pseudotyped HIV-CMV-DTA were not subjected to flow cytometric analysis due to the lack of live cells present within the co-culture (Fig. 7c, bottom panel). Thus, these data demonstrate that the VSV-G-pseudotyped particles are able to target diverse cell types due to use of a ubiquitously expressed cell surface receptor, while the MLV Env-pseudotyped particles are only able to target cells expressing the mCAT-1 receptor.

## Discussion

Diphtheria toxin, made naturally by *Corynebacterium diphtheria*, is a secreted polypetide that is cleaved into A and B fragments upon entry into eukaryotic cells. The B chain is responsible for binding of the toxin to markers present on the cell surface, while the A chain is responsible for the catalytic activity of the toxin and inhibition of protein synthesis in the target cell^4,7^. Notably, DTA released from dead cells is not able to enter bystander cells, due to the lack of the cell-targeting B chain.

Use of DTA has been explored in a variety of potentially therapeutic systems, including those that target both HIV and various cancers. For example, a non-integrating, Rev-dependent lentiviral vector encoding DTA and human TRAF6 was used to target HIV reservoirs^18^. In the case of this vector, expression is entirely dependent upon expression of Rev, which would be expressed only in HIV-infected cells. Other work has demonstrated regression of prostate cancer xenografts by a lentiviral vector expressing DTA under control of a prostate cancer-specific promoter^20^.

Here, we engineered a lentiviral vector expressing DTA under control of the CMV promoter for use in *in vitro* negative selection strategies. Utility of this DTA-expressing vector could apply to a variety of experimental strategies, such as those employing genome-wide CRISPR/Cas9 screening to identify cells resistant to infection by the lentiviral vector, those examining mutagenized envelope glycoproteins to ascertain compatibility with a variety of cell types, or those to identify yet unknown envelope glycoprotein receptors and co-receptors. To allow robust production of lentiviral particles expressing the DTA transgene and evaluation of DTA-induced effects in target cells, we engineered DTA-resistant producer and target cells through CRISPR/Cas9-mediated knockout of the DPH1 gene. DPH1 is a component of a multi-step pathway for diphthamide synthesis^7,28^. Diphthamide is an unusual modified histidine residue in eEF2, and is the target of the catalytic activity of DTA. ADP-ribosylation of diphthamide by DTA inhibits eEF2 function by blocking protein synthesis^5,6,28^.

Our results demonstrate that DTA encoded by our lentiviral vector is functional in the context of transfection of the proviral plasmid (Fig. 3) and transduction of the lentiviral particles into target cells (Fig. 4). Importantly, the vector can be specifically targeted to cells expressing mCAT-1 via pseudotyping of the lentiviral vector with MLV Env (Fig. 7). Numerous other viral glycoproteins could also be used for cell-specific targeting. DTA produced upon transduction of our lentiviral vector into target cells was able to induce cell death in target cells. Notably, the effect was DTA-specific, as target cells modified to be resistant to DTA-induced effects through knockout of DPH1 were infected, but remained viable (Fig. 6). Thus, the lentiviral vector described here, expressing DTA under control of the constituitive CMV promoter, will be a useful tool for *in vitro* negative selection experiments. Importantly, the only modification required will be selection of a specific, cell-targeting viral glycoprotein for pseudotyping.

## Methods

Unless otherwise noted, all chemicals were purchased from Sigma-Aldrich (St. Louis, MO). Restriction enzymes used for cloning purposes were purchased from New England Biolabs (Ipswitch, MA). The InFusion HD cloning kit was purchased from Takara Bio USA, Inc. (Mountain View, CA). Primers were purchased from Integrated DNA Technologies (Coralville, IA).

### Plasmids

The HIV-1_NL3-4_-derived plasmid, modified for single cycle infectivity assays and referred to herein as pHIV-CMV-EGFP, was kindly provided by Vineet KewalRamani (National Cancer Institute, Fredrick, MD). This proviral vector lacks the genes encoding *vif*, *vpr*, *vpu*, *nef*, and *env*, and has a CMV immediate early promoter-driven EGFP in place of *nef*. The pHIV-CMV-EGFP vector was further modified to restore the *vpu* sequence^33^, and to replace the EGFP with the sequence for diphtheria toxin A (pHIV-CMV-DTA, Fig. 1), amplified from a DTA-expressing plasmid kindly provided by Mark Garcia, University of Missouri. DTA was amplified using the following primers: 5′-AACCGTCAGATCCGCTAGCCACCATGGATCCTGATGATGTTGTTGCGGCCGCTTTAGAGCTT-3′ and 5′-ATGTTTTTCTAGGTCTCGAGATTAGAGCTTTAAATCTCTGTAG-3′. The DTA amplicon was inserted using InFusion Cloning (Takara Bio USA Inc, Mountain View, CA) into the pHIV-CMV-EGFP vector digested with XhoI and NheI for removal of the EGFP sequence. The vesticular stomatitis virus (VSV) glycoprotein-expressing plasmid used for viral pseudotyping, referred to herein as pVSV-G, was obtained from Invitrogen (pMD-G, Carlsbad, CA). The plasmid encoding the Murine Leukemia Virus envelope glycoprotein (MLV Env) was kindly provided by Walter Mothes, Yale University. The lentiCRISPRv2 lentiviral vector and psPAX2 packaging vector were obtained from Addgene (Cambridge, MA). GFP-N1, referred to herein as pCMV-EGFP, was obtained from Clontech (now Takara Bio USA, Inc, Mountain View, CA). pCMV-mCherry was engineered by replacing the EGFP sequence in pCMV-EGFP with the mCherry sequence amplified from pNCS mCherry, a generous gift from the Erik Rodriguez and Roger Tsien^34^. Primers used for amplification of mCherry were 5′-GTGTGTAGATCTCATGGTGAGCAAGGGCGAG-3′ and 5′-ATATATGCGGCCGCTCGAGTTACTTGTACAGCTCGTCC-3′. The EGFP gene was removed using XbaI and NotI restriction sites.

### Cell lines and viruses

The human cell lines, HEK 293FT (Invitrogen, Carlsbad, CA), TZM-GFP^25^, 293T 5H7 (Kerafast, Boston, MA)^18^, 293mCAT-1 cells (Walter Mothes, Yale University), and modified cell lines 293FT-DPH1KO and TZM-GFP-DPH1KO, were maintained in standard culture media containing Dulbecco’s Minimum Essential Medium (Sigma, St. Louis, MO), 10% FBS (Sigma, St. Louis, MO), 2 mM L-glutamine (Gibco, Life Technologies, Grand Island, NY), 1 mM non-essential amino acids (Gibco, Life Technologies, Grand Island, NY), and 1 mM sodium pyruvate (Gibco, Life Technologies, Grand Island, NY). All cell lines were maintained at 37 °C in 5% carbon dioxide. Cells were split using TryplExpress (Gibco, Life Technologies, Grand Island, NY) at least twice per week.

Modified cell lines, 293FT-DPH1KO and TZM-GFP-DPH1KO were generated via transduction with the lentiCRISPRv2 lentiviral vector modified to express a CRISPR guide RNA sequence targeting the DPH1 gene (GTTCACGGAGGCCGAAGTGA) obtained from the GeCKO library sequence bank, SpCas9, and a puromycin resistance cassette^27^. Lentiviral vectors were generated by polyethylenimine (PEI, Sigma, St. Louis, MO) transfection of 293FT cells with the lentiviral vector, packaging vector psPAX2, and pVSV-G. Vectors were then used to transduce 293FT and TZM-GFP cells. After 48 hours, transduced cells were selected with puromycin. Puromycin-resistant populations were then seeded to obtain single cell isolates, each of which were tested for specific insertion/deletion modifications of the DPH1 target by PCR and sequencing, and for resistance to DTA expression in cell culture by transfection as described below. Primers used for validation of both the DPH1 sgRNA sequence and DPH1 target site are provided in Fig. S1.

Parental (HIV-CMV-EGFP) and DTA-encoding lentiviruses were generated via PEI-mediated co-transfection of 293FT and 293FT-DPH1KO cells or jetPRIME (Polyplus, New York, NY)-mediated co-transfection of TZM-GFP and TZM-GFP-DPH1KO cells with pHIV-CMV-EGPF or pHIV-CMV-DTA and the specified viral glycoproteins for pseudotyping. Virus-containing supernatant was harvested after 48 hours and centrifugation was performed to remove cellular debris. Transduction efficiencies for DTA-encoding viruses pseudotyped with pVSV-G were obtained on TZM-GFP and TZM-GFP-DPH1KO cells. Viral titers for all viruses were obtained on TZM-GFP-DPH1KO cells. Viruses were stored at −80 °C.

### Analysis of CRISPR/Cas9 modifications to DPH1

Genomic DNA was isolated from parental and puromycin-resistant populations, as well as single cell isolates for each cell type using the DNeasy Blood and Tissue Kit (Quiagen, Germantown, MD). After DNA isolation, the presence of the DPH1 sgRNA sequence and modification at the DPH1 target site were determined by amplification of the vector cloning site (for sgRNA sequence confirmation) or a specific portion of the DPH1 gene spanning the target site. Primer sequences used for amplification of the DPH1 target gene can be found in Fig. S1. Following PCR amplification, PCR products were gel extracted using the NucleoSpin Gel and PCR Cleanup kit (Takara Bio USA, Mountain View, CA) and submitted for Sanger Sequencing Analysis at the University of Missouri DNA Sequencing Core. Sequencing results were visualized using SnapGene software (GSL Biotech LLC, Chicago, IL). Target site modifications were analyzed using CRISPR TIDE (https://tide.nki.nl/) and chromatogram analysis to determine the specific location of insertions or deletions in the CRISPR/Cas9-generated, monoclonal knockout cell lines^29^. Target site modifications can be found in Fig. S1.

### DTA inhibition of protein translation

The 293FT, 293FT-DPH1KO, TZM-GFP and/or TZM-GFP-DPH1KO cells were plated in 6 well plates. The next day, cells were co-transfected with a combination of either pCMV-EGFP (25 ng), pUC19 (1000 ng), and pVSV-G (100 ng), or pCMV-EGFP (25 ng), pHIV-CMV-DTA (1000 ng), and pVSV-G (100 ng). After 48 hours, fluorescent images were taken using an Olympus XI81 microscope (Olympus Scientific Solutions, Waltham, MA), supernatant was harvested from each well, and cell debris was removed by centrifugation. Cells were washed with 1X PBS, collected via trypsinization, fixed with 2% paraformaldehyde, and washed with 1X PBS. Cells were analyzed on an Accuri B6 Flow Cytometer (BD Biosciences, San Jose, CA) to determine the percentage of GFP-positive cells, indicating the status of EGFP protein translation in the cells.

### Infectivity assays

The 293FT, 293FT-DPH1KO, TZM-GFP and/or TZM-GFP-DPH1KO cells were plated in 6 well plates. The next day, cells were co-transfected with a combination of either pCMV-EGFP (25 ng), pUC19 (1000 ng), and pVSV-G (100 ng), or pCMV-EGFP (25 ng), pHIV-CMV-DTA (1000 ng), and pVSV-G (100 ng). Plasmid pCMV-EGFP was included to monitor DTA resistance (via inhibition of EGFP protein translation) or susceptibility in the cell lines. For a subset of the assays in TZM-GFP and TZM-GFP-DPH1KO cells, pVSV-G was replaced with a plasmid encoding the MLV Envelope glycoprotein (MLV Env). In parallel, for comparison of virus yield, 293FT-DPH1KO or 293T 5H7 cells were co-transfected with either pHIV-CMV-EGFP or pHIV-CMV-DTA and pVSV-G for comparison of DTA-encoding and non-DTA-encoding viruses. After 48 hours, supernatant was harvested from each well and cell debris was removed by centrifugation. Viral titers were obtained on TZM-GFP-DPH1KO cells via serial dilution and counting of GFP-positive cells at each dilution. Viruses were then used to infect specified target cells with the indicated volume of supernatant or MOI. Images were taken at indicated time points post-infection using an Olympus XI81 microscope (Olympus Scientific Solutions, Waltham, MA). Following microscope analysis, when applicable, cells were washed with 1X PBS, collected via trypsinization, fixed with 2% paraformaldehyde, and washed with 1X PBS. Cells were analyzed on an Accuri B6 Flow Cytometer (BD Biosciences, San Jose, CA) to determine the percentage of GFP-positive cells, indicating viral infectivity.

### Cell death assays

DTA-encoding lentiviruses were produced in 293FT-DPH1KO cells as described above using the pVSV-G glycoprotein for pseudotyping. Viruses were serially diluted and titered on TZM-GFP and TZM-GFP-DPH1KO cells by counting the number of GFP-positive foci at each dilution. For initial cell death assays, TZM-GFP and TZM-GFP-DPH1KO cells were plated in 96 well plates at a density of 5000 cells per well and were infected with DTA-encoding viruses at an MOI of >1. For cell targeting death assays, the indicated cell lines were plated in 96 well plates at a density of 5000 cells per well, or at a 1:1 ratio, and infected with DTA-encoding viruses at an MOI > 1. After 96 hours, 96 well plate experiments were subjected to crystal violet staining and brightfield imaging using an Olympus IX81 microscope (Olympus Scientific Solutions, Waltham, MA). For crystal violet staining, cells were gently washed twice with 1X PBS and subsequently incubated with 0.5% crystal violet staining solution containing methanol fixative for 20 minutes. Following staining, cells were again gently washed with 1X PBS and dried for imaging.

## Supplementary information

Supplementary Dataset 1

## Data Availability

All materials, data, and associated protocols will be available upon request.
