# Supplementary material for: Diphtheria Toxin A-Resistant Cell Lines Enable Robust Production and Evaluation of DTA-Encoding Lentiviruses
Source: Sci Rep. 2019 Jun 20;9:8985. doi: 10.1038/s41598-019-45481-9 (PMC6586843; doi:10.1038/s41598-019-45481-9)
Supplement: Supplementary file 1 — Supplementary Dataset 1 [file 41598_2019_45481_MOESM1_ESM.docx]

Title: Diphtheria Toxin A-Resistant Cell Lines Enable Robust Production and Evaluation of DTA-Encoding Lentiviruses

Margaret J. Lange^1,2*^, Terri D. Lyddon^2^, and Marc C. Johnson^1,2*^.

Department of Molecular Microbiology and Immunology^1^, Bond Life Sciences Center^2^, University of Missouri, Columbia, Missouri, USA.

*Corresponding Authors

To whom correspondence may be addressed:

Email: [langemj@missouri.edu](mailto:langemj@missouri.edu) or [marcjohnson@missouri.edu](mailto:marcjohnson@missouri.edu)

Present Address: Margaret Lange or Marc Johnson, Department of Molecular Microbiology & Immunology, Life Sciences Center, University of Missouri, Columbia, MO, 65211, United States

**Supplementary Figure 1**

**
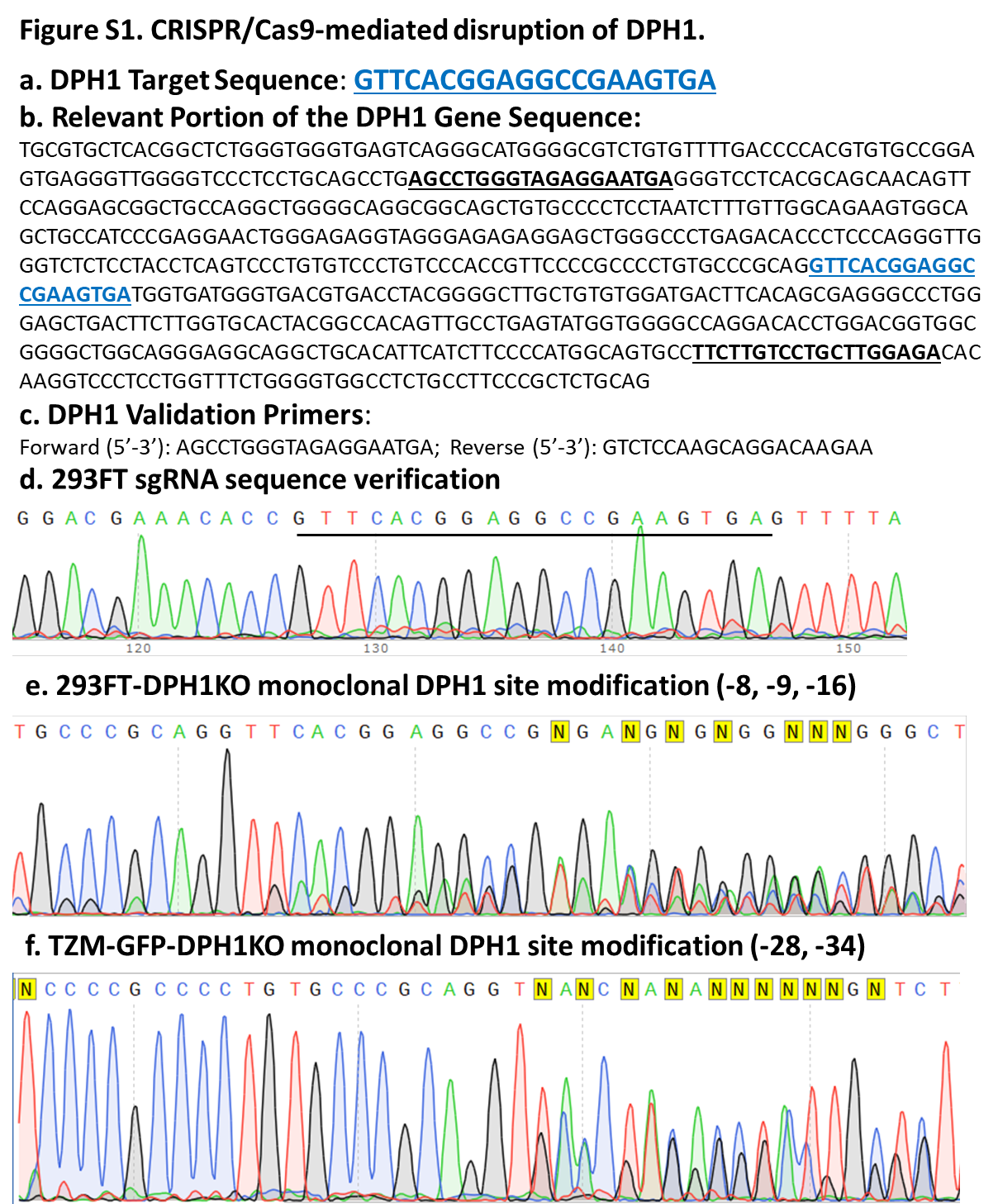
**

**Supplementary Figure Legend**

**Figure S1. DPH1 target site location and sequence modifications.** (a) DPH1 target sequence. (b) Location of DPH1 target sequence within the DPH1 gene. (c) Primer sequences used for validation of DPH1 target site modification within the DPH1 gene. (d) Sequencing result demonstrating presence of the DPH1 guide sequence within the lentiviral vector construct. (e and f) Sequencing result demonstrating modification of the DPH1 target site within the cellular genomic DNA for 293FT-DPH1KO and TZM-GFP-DPH1KO.
